# Clinical characteristics and outcomes in febrile infants aged 29–90 days with urinary tract infections and cerebrospinal fluid pleocytosis

**DOI:** 10.3389/fped.2023.1196992

**Published:** 2023-05-30

**Authors:** Ga Won Moon, Donghyun Shin, Young Mi Kim, Soo-Han Choi

**Affiliations:** ^1^ Department of Pediatrics, Pusan National University Hospital, Busan, Republic of Korea; ^2^ Department of Pediatrics, Pusan National University School of Medicine, Busan, Republic of Korea; ^3^ Biomedical Research Institute, Pusan National University Hospital, Busan, Republic of Korea

**Keywords:** infant, urinary tract infections, bacterial meningitis, cerebrospinal fluid pleocytosis, fever without a focus

## Abstract

**Introduction:**

Fever without a focus is a common reason for medical evaluations, hospitalizations, and the antimicrobial treatment of infants younger than 90 days. The presence of cerebrospinal fluid (CSF) pleocytosis could be challenge for clinicians who treat febrile young infants with urinary tract infection (UTI). We evaluated the factors associated with sterile CSF pleocytosis and the clinical outcomes of the patients.

**Methods:**

A retrospective review of patients aged 29–90 days with febrile UTIs who underwent a non-traumatic lumbar puncture (LP) at Pusan National University Hospital from January 2010 to December 2020 was conducted. CSF pleocytosis was defined as white blood cell (WBC) counts ≥9/mm^3^.

**Results:**

A total of 156 patients with UTI were eligible for this study. Four (2.6%) had concomitant bacteremia. However, no patients had culture-proven bacterial meningitis. In correlation analysis, although weak strength, CSF WBC counts were positively correlated with C-reactive protein (CRP) level (Spearman *r* = 0.234; *P* = 0.003). Thirty-three patients had CSF pleocytosis [21.2%; 95% confidential interval (CI), 15.5–28.2]. The time from fever onset to the hospital visit, peripheral blood platelet counts, and CRP level at admission were statistically significant in patients with sterile CSF pleocytosis compared to those without CSF pleocytosis. In the multiple logistic regression, only CRP was independently associated with sterile CSF pleocytosis (cutoff, 3.425 mg/dl; adjusted odds ratio, 2.77; 95% CI, 1.19–6.88). The proportion of fever defervescence by hospital day 2 was 87.9% in patients with CSF pleocytosis and 89.4% in those without CSF pleocytosis (*P* = 0.759). There was no statistical difference in the fever defervescence curves between the two patient groups (*P *= 0.567). No patients had neurological manifestations or complications.

**Conclusions:**

Coexisting sterile CSF pleocytosis among febrile infants with UTIs suggest a systemic inflammatory response. However, the clinical outcomes between the two groups were similar. A selective LP should be considered in young infants with evidence of UTI, and inappropriate antibiotic therapy for sterile CSF pleocytosis should be avoided.

## 1. Introduction

Fever without a focus is a common reason for medical evaluations, hospitalizations, and the antimicrobial treatment of infants younger than 90 days (1). Serious bacterial infections occur in 7%–13% of neonates and young infants with fever. In febrile young infants, urinary tract infections (UTIs) are the most common bacterial infections (5%–13%), and the reported prevalence of bacteremia associated with UTI ranges from 4% to 10% (1–4). In theory, young infants with bacteremia are at risk for the hematogenous dissemination of pathogenic bacteria to the central nervous system (2). However, previous studies found that the risk of bacterial meningitis associated with UTI was low in febrile young infants (5–20). In a systematic review and meta-analysis, the prevalence of bacterial meningitis among infants aged 29–60 days with positive urinalysis results ranged from 0.25% to 0.44% (18).

Despite the low prevalence of coexisting bacterial meningitis, the majority of febrile young infants undergo lumbar puncture (LP) to obtain cerebrospinal fluid (CSF) samples regardless of the urinalysis results. The need for LP among young infants with a presumptive UTI has been questioned for decades (4, 12, 21, 22). LP is an invasive procedure and is not always successful in young infants. The rate of failure or traumatic LP in infants younger than 90 days was reported to range from 20% to 50% (4). Furthermore, sterile CSF pleocytosis is not infrequently observed in young infants with febrile UTIs who undergo LP, occurring in 18%–29% of patients in several studies (13, 15). The presence of CSF pleocytosis can be another challenge for clinicians who treat febrile young infants with abnormal urinalysis results, and those with CSF pleocytosis are likely to receive prolonged intravenous or unnecessary additional antibiotic therapies, even after culture-proven UTIs and negative CSF culture results are confirmed (7, 13, 20). However, very few studies on the clinical predictors of sterile CSF pleocytosis or clinical outcomes in this population have been conducted (7, 13, 14). This information could help the clinician in decision-making regarding performing LP and appropriate management for febrile infants aged 29–90 days with abnormal urinalysis results.

The aims of this study were to investigate the prevalence of bacterial meningitis and sterile CSF pleocytosis among febrile infants 29–90 days of age with UTIs. In addition, we evaluated the factors associated with sterile CSF pleocytosis in those patients and compared the clinical outcomes according to the presence of sterile CSF pleocytosis.

## 2. Materials and methods

### 2.1. Study patients and definition

A retrospective, cross-sectional study was conducted at a tertiary-care university hospital, Pusan National University Hospital, the Republic of Korea. We investigated febrile infants aged 29–90 days who simultaneously underwent urinalysis with urine culture, blood culture, and LP to evaluate fever without a focus between January 2010 and December 2020. Of these, infants diagnosed with UTI were eventually included in this study. The presence of fever was defined as axillary or tympanic temperature ≥38°C in the hospital or at home within 24 h. Infants with unstable vital signs or specific symptoms and signs other than fever at the hospital visit, those receiving antibiotics in the preceding 48 h before cultures acquisition, and those with traumatic LP (defined as ≥30 red blood cells/mm^3^ of CSF specimen) were excluded from this study.

UTI was defined as the growth of a single urine pathogen with ≥50,000 colony-forming units/mL from catheterized specimens in association with an abnormal urinalysis [>5 white blood cells (WBCs) per high powered field, or positive leukocyte esterase, or nitrite] (23). Bacterial meningitis was defined as the growth of a single bacterial pathogen from the CSF specimen. CSF pleocytosis was defined as ≥9 WBC/mm^3^ using age-specific CSF WBC reference values (24). Concomitant bacterial meningitis or bacteremia was defined as a positive CSF or blood culture with the growth of the same bacterial pathogen recovered from the urine. The following bacteria were classified as contaminants: *Bacillus, Corynebacterium, Micrococcus, Propionibacterium*, and coagulase-negative *Staphylococcus*.

### 2.2. Data collection

We collected data on patient demographics, past medical history, onset time of fever, and general appearance of the patients (presence of decreased activity, inconsolability, mottling, or cyanosis) (25) at the initial visit. The laboratory data included the following: peripheral blood WBC count, absolute neutrophil count (ANC), platelet count, C-reactive protein (CRP), urinalysis, CSF analysis (cell counts, protein, and glucose), and bacterial culture results (urine, blood, and CSF). The results of imaging studies such as renal and bladder ultrasonography (RBUS) or renal scintigraphy were also collected. The clinical outcome variables were fever defervescence by hospital day 2, length of hospital stay, and neurological complications. The defervescence day was defined as the hospital day when the last fever occurred. We compared demographic data, characteristics, and clinical outcomes according to the presence of CSF pleocytosis or concomitant bacterial meningitis.

### 2.3. Statistical analysis

We used descriptive statistics, including medians, interquartile ranges (IQRs), counts, and proportions with 95% confidence intervals (95% CIs). We divided the patients into two groups according to the presence of sterile CSF pleocytosis, and compared the clinical variables. Categorical variables were compared by Fisher's exact test. Continuous variables were compared by the Mann–Whitney *U* test. Fever defervescence curves according to hospital day between the two groups were compared by the log-rank test. There were no missing data.

The correlation between continuous variables and CSF WBC counts was performed by Spearman's correlation analysis. Multiple logistic regression analysis was performed to assess the independent factors associated with sterile CSF pleocytosis, and adjusted odds ratios (aORs) and 95% CIs were calculated. We chose potential predictors using statistically significant variables in univariable analysis and included inflammatory markers, regardless of statistical significance. We determined the cutoff values that maximized the area under the receiver operating characteristic curve. We tested for multicollinearity with variance inflation factors and performed the Hosmer-Lemeshow test for goodness-of-fit. Two-sided *P*-values of <0.05 were considered statistically significant. Data were analyzed using Prism 9.3.1 (GraphPad Software Inc., San Diego, CA, USA).

## 3. Results

### 3.1. Patients characteristics

**Figure 1:**
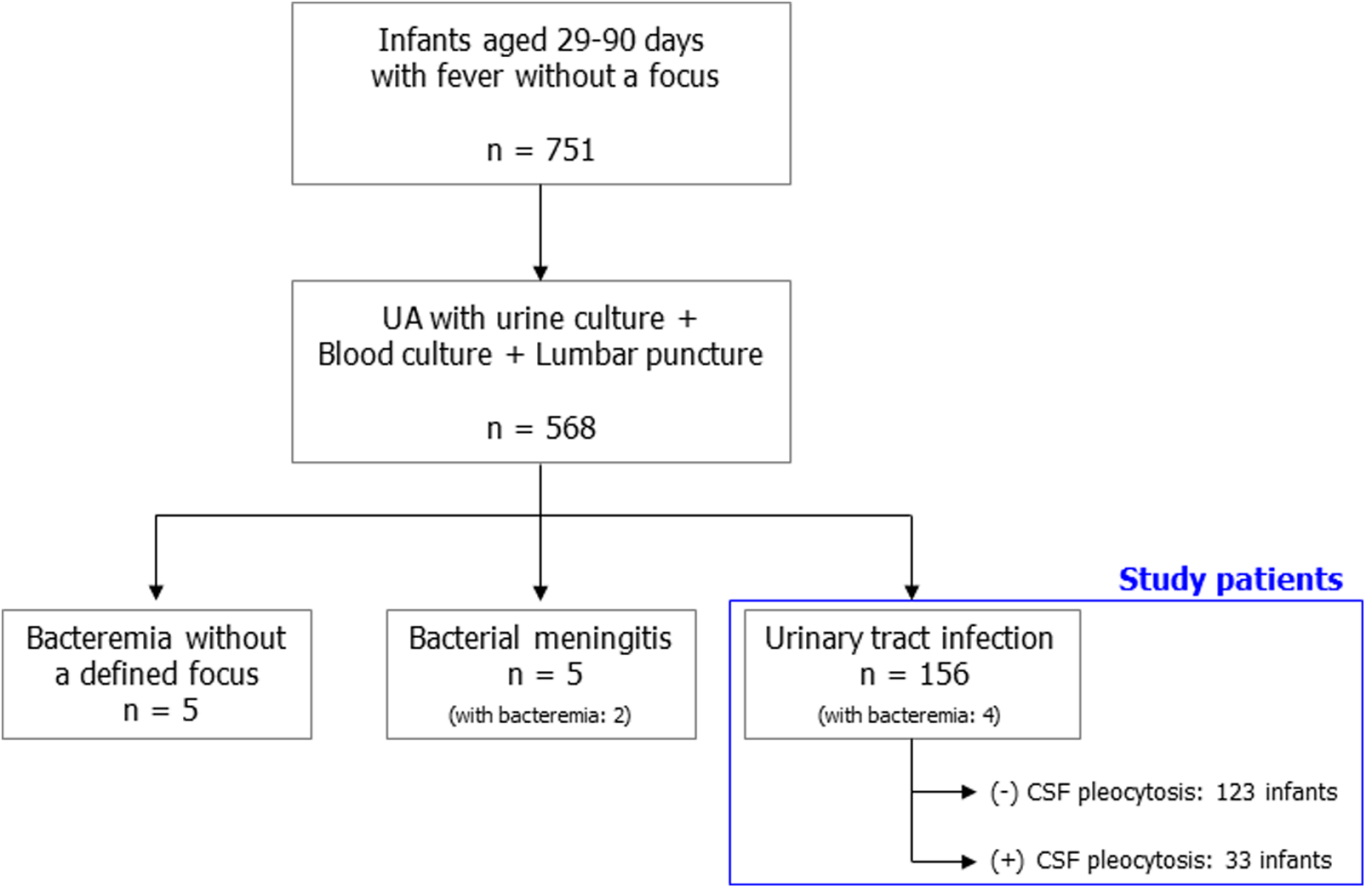
Flowchart of the study patients.

During the study period, 751 infants aged 29–90 days had fever without a focus. Of these, 568 patients received urinalysis with urine culture, blood culture, and LP at the same time. A total of 156 patients with febrile UTIs were finally eligible for this study (Figure 1). The characteristics of the study patients are summarized in Table 1. Three-fourths of the patients visited the hospital within 24 h of fever onset. The median age of the patients was 62 days (IQR, 48–72 days), and the proportion of infants aged 29–60 days was 46.2% (72/156). All patients were hospitalized and received parenteral antibiotic therapy. During hospitalization, all patients underwent one or more imaging studies for their UTI (RBUS, 156; renal scintigraphy, 97). Forty-four (28.2%) had grade 3 or higher hydronephrosis or acute pyelonephritis. The most frequently isolated urine pathogen was extended-spectrum beta-lactamase (ESBL) negative *Escherichia coli* (73.1%; 114). Concomitant bacteremia was observed in four infants (2.6%, 4/156): 4.2% (3/72) of infants aged 29–60 days; 1.2% (1/84) of infants aged 61–90 days. However, no patients had culture-proven bacterial meningitis. Enterovirus PCR or multiplex PCR for microbial detection in CSF specimens was performed in 135 patients (86.5%). Enterovirus positivity was observed in one patient whose CSF WBC count was 1/mm^3^.

**Table 1:** Characteristics of study patients.

	*N* = 156
Age, days, median (range)	62 (29–90)
Sex, male (%)	129 (82.7)
Time from fever onset to the hospital visit, median (interquartile range), hours	9 (4–21)
Abnormality in the imaging studies^a^ (%)	44 (28.2)
Bacteremia^b^ (%)	4 (2.6)
**Isolated urinary pathogens (%)**	
ESBL (+) *E. coli*	18 (11.5)
ESBL (−) *E. coli*	114 (73.1)
ESBL (+) *Klebsiella* species	0 (0)
ESBL (−) *Klebsiella* species	13 (8.3)
*Enterobacter* species	6 (3.8)
Others	5 (3.2)
**Cerebrospinal fluid analysis**	
White blood cell counts, cells/mm^3^	
Median (range)	4 (0–91)
Interquartile range	2–8
Protein, median (range), mg/dl	43.85 (19.7–111.5)
Glucose, median (range), mg/dl	61 (43–132)
Cerebrospinal fluid culture positive	0

^a^ Grade 3 or higher hydronephrosis or acute pyelonephritis on renal and bladder ultrasonography or renal scintigraphy.

^b^ ESBL (-) *Escherichia coli* (3); *Enterobacter aerogenes* (1). *E. coli, Escherichia coli*; ESBL, extended-spectrum beta-lactamase.

### 3.2. CSF pleocytosis

**Figure 2:**
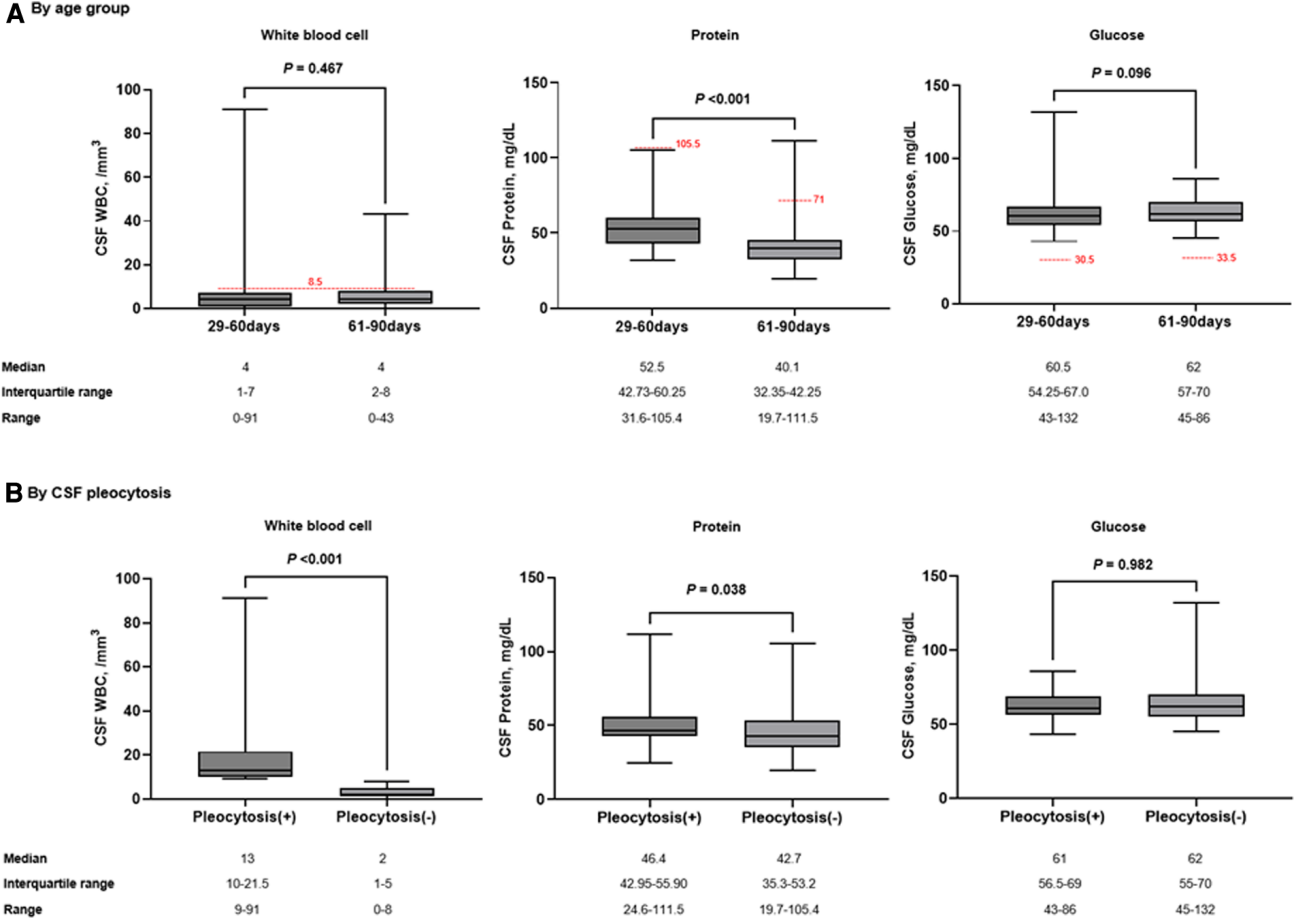
Comparison of CSF parameters by age group (**A**) and the presence of CSF pleocytosis (**B**). The red dotted horizontal lines indicate the upper limit of normal by age-specific reference. Box indicates median value and quartiles. Whiskers indicate the minimum and maximum values.

Of all patients, the median CSF WBC count was 4/mm^3^ (range, 0–91). There was no difference in CSF WBC counts between infants aged 29–60 days and 61–90 days (Figure 2A). In the correlation analysis, although weak strength, CRP was positively correlated with CSF WBC counts (Spearman *r* = 0.234; *P* = 0.003) (Supplementary Table S1). Sterile CSF pleocytosis was observed in 33 patients (21.1%; 95% CI, 15.5–28.2): 18.1% (13/72; 95% CI, 10.9–28.5) in infants aged 29–60 days and 23.8% (20/84; 95% CI, 16.0–33.9) in those aged 61–90 days (*P* = 0.435). For patients with CSF pleocytosis, the median CSF protein value was higher than in those without CSF pleocytosis (Figure 2B). Among patients with CSF pleocytosis, two patients had a higher CSF protein value than the upper limit of normal according to the age-specific reference. One patient had concomitant bacteremia with CSF protein and glucose values within the normal ranges.

### 3.3. Factors associated with the presence of sterile CSF pleocytosis

A comparison of the clinical characteristics at admission according to the presence of sterile CSF pleocytosis was described in Table 2. Compared to patients without CSF pleocytosis, patients with CSF pleocytosis had longer time from fever onset to the hospital visit (median, 15 vs*.* 8 h; *P *= 0.037) and higher platelet counts (median, 461,000 vs*.* 399,000/mm^3^; *P* = 0.020) and CRP values (median, 4.27 vs*.* 2.48 mg/dl; *P* = 0.012). There were no significant differences in general appearances at the initial visit, the degree of pyuria, peripheral blood WBC counts, and ANCs. In patients with CSF pleocytosis, the proportion of grade 3 or higher hydronephrosis was higher but not statistically significant (12.1% vs*.* 4.1%; *P* = 0.095).

**Table 2:** Comparison of characteristics according to the presence of CSF pleocytosis.

	Pleocytosis (+) *N* = 33	Pleocytosis (−) *N* = 123	*P*-value
Age, days, median (range)	63 (33–89)	61 (29–90)	0.711
Age 29–60 days (%)	13 (39.4)	59 (48.0)	0.435
Sex, male (%)	31 (93.9)	98 (79.7)	0.069
Premature [gestational age <37 weeks] (%)	1 (3.0)	7 (5.7)	>0.999
Summer season [June-August] (%)	10 (30.3)	44 (35.8)	0.681
Not well appearance^a^ at the initial visit (%)	18 (54.6)	60 (48.8)	0.695
Time from fever onset to the hospital visit Median (range), hours	15 (1–120)	8 (1–96)	0.037
Temperature >38.5°C before or at the hospital visit (%)	15 (45.4)	72 (58.5)	0.236
**Urinalysis (%)**			
WBC counts, ≥30/HPF	22 (66.7)	93 (75.6)	0.373
Nitrite (+)	16 (48.5)	48 (39.0)	0.426
**Laboratory findings on admission**			
White blood cell, /mm^3^, median (range)	16,450 (4,230–25,740)	14,440 (2,780–30,820)	0.455
ANC, /mm^3^, median (range)	6,847 (1,936–16,988)	6,421 (1,109–19,970)	0.622
Platelet, ×10^3^/mm^3^, median (range)	461 (233–994)	399 (108–1,000)	0.020
C-reactive protein, mg/dl, median (range)	4.27 (0.15–28.46)	2.48 (0.0–15.11)	0.012
Bacteremia (%)	1 (3.0)	3 (2.4)	>0.999
Hydronephrosis ≥grade 3 (%)	4 (12.1)	5 (4.1)	0.095

^a^ Presence of decreased activity, inconsolability, mottling, or cyanosis.

HPF, high power field; ANC, absolute neutrophil count.

The time from fever onset to the hospital visit, peripheral blood WBC count, ANC, platelet count, and CRP vale were determined to be potential predictors associated with the presence of CSF pleocytosis. The cutoff values for the laboratory values were as follows: peripheral blood WBC count of 15,080/mm^3^, ANC of 6,710/mm^3^, platelet count of 415,500/mm^3^, and CRP of 3.425 mg/dl (Supplementary Figure S1). In the multiple logistic regression analysis (Table 3), only CRP was an independent factor associated with sterile CSF pleocytosis (aOR, 2.77; 95% CI, 1.19–6.88; *P* = 0.019).

**Table 3:** Factors associated with the presence of sterile CSF pleocytosis at the initial diagnosis.

Variables	Adjusted odds ratio (95% CI)	*P*-value
Time from onset of fever to hospital visit ≥12 h	1.31 (0.56–3.03)	0.533
White blood cell >15,080/mm^3^	1.32 (0.45–3.89)	0.606
Absolute neutrophil counts >6,710/mm^3^	1.03 (0.35–2.97)	0.958
Platelet >415,500/mm^3^	1.92 (0.84–4.49)	0.122
C-reactive protein >3.425 mg/dl	2.77 (1.19–6.68)	0.019

CI, confidential interval; hrs, hours. Multiple logistic regression analysis was performed with the Hosmer-Lemeshow test (*P* = 0.984) and the variance inflation factor of all variables was between 1 and 2.

### 3.4. Clinical outcomes

**Figure 3:**
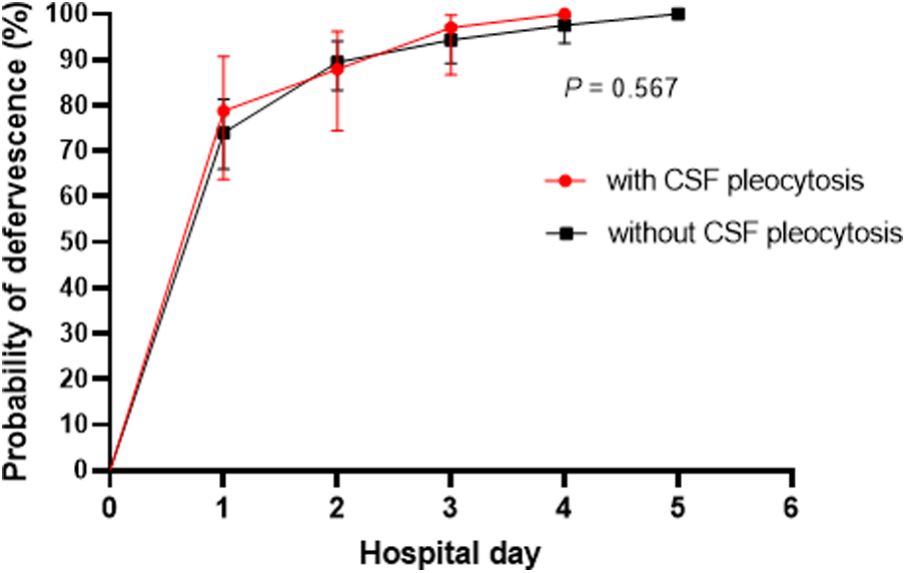
Comparison fever defervescence curves between the patients with and without CSF pleocytosis. The vertical bars indicate 95% confidence intervals.

There was no significant difference in defervescence after hospitalization according to the presence of CSF pleocytosis. Fever subsided within 24 h in the majority of the patients. The proportion of patients with fever defervescence by hospital day 2 was 87.9% in those with CSF pleocytosis and 89.4% in those without CSF pleocytosis (*P* = 0.759). There was no statistical difference in defervescence curves between the two patient groups (Figure 3; hazard ratio, 1.06; 95% CI, 0.72–1.57; *P* = 0.567). The median duration of hospitalization was 7 days (range, 4–21) in all patients, and there was no difference between the two groups. Among all study patients, no patients had neurological manifestations during hospitalization or complications after hospital discharge.

## 4. Discussion

In our study of 156 infants aged 29–90 days with febrile UTIs, no patient had concomitant bacterial meningitis. Sterile CSF pleocytosis was observed in 21.2% (29–60 days, 18.1%; 61–90 days, 23.8%). CRP was the only independent factor associated with CSF pleocytosis. Clinical outcomes were similar regardless of CSF pleocytosis, and no patients had neurological complications.

Consistent with our findings, several recent studies showed that the prevalence of bacterial meningitis in young febrile infants with positive urinalysis results was very low. A systematic review and meta-analysis of 20 studies noted that the pooled prevalence of bacterial meningitis was 0.25% (95% CI, 0.09–0.70) among 3,868 infants aged 29–90 days with evidence of UTI (18). In another systematic review and meta-analysis of 17 distinct international data-sets, the prevalence of culture-proven meningitis was 0.44% (95% CI, 0.25–0.78) among 2,703 infants aged 29–60 days with positive urinalysis results. This prevalence was not higher than in infants with negative urinalysis results (0.50%; 95% CI, 0.33–0.76) (17). Mahajan et al. explored the risk of bacteremia or meningitis in non-toxic appearing febrile infants ≤60 days old with positive urinalysis results (19). The risk of bacteremia was greater in those with positive urinalysis results compared to negative urinalysis results [5.8% (63/1,090) vs*.* 1.1% (69/6,090)], but there was no difference in meningitis risk between the two groups (0.4% vs*.* 0.6%). No cases of bacterial meningitis (0/697) were found in infants 29–60 days old with positive urinalysis results, whereas 0.2% of those with negative urinalysis had meningitis (9/4,153). These studies suggest that having a UTI does not increase the probability of bacterial meningitis, and LP is not essentially needed in the evaluation of fever in young infants with a presumptive UTI beyond the neonatal period. The American Academy of Pediatrics (AAP) guidelines recommend performing LP on well-appearing febrile infants 29–60 days old with positive urinalysis results, if any inflammatory markers level are abnormal (procalcitonin >0.5 ng/ml or fever of >38.5°C in combination with CRP ≥ 20 mg/L or ANC > 4000, >5200 per mm^3^) (4). Among febrile infants aged 29–60 days with UTI in our study, the proportions of patients with abnormal inflammatory markers in the AAP guidelines (fever of >38.5°C in combination with CRP ≥ 20 mg/L or ANC > 5200 per mm^3^) were similar between well-appearing term infants and the other infants (40.0% vs*.* 45.2%, *P* = 0.810; Supplementary Table S2). However, our study patients were heterogenous, and the prevalence of bacteremia in our study was lower than in previously reported studies. In addition, although we evaluated general appearance based on the pediatric assessment triangle (25), the possibility of subjective judgment could not be ruled out.

Although UTI is rarely associated with bacterial meningitis, sterile CSF pleocytosis is relatively common in young infants with UTIs who undergo LP (6–11, 13, 15). In a systematic review, the prevalence of sterile CSF pleocytosis in infants aged 29–90 days with evidence of UTI ranged from 0% to 29% (17). Direct comparisons with this study are limited because the definition of CSF pleocytosis, the inclusion or exclusion of traumatic LP, the correction of traumatic LP, and the range of ages varied between studies. Thomson et al. used the same definition of CSF pleocytosis as this study (≥9 WBC/mm^3^) but made no mention of traumatic LP. The prevalence of CSF pleocytosis was 29.1% (95% CI, 26.0–32.4) in infants aged 29–60 days (15). In Adler-Shohet et al., the rate of CSF pleocytosis among infants aged 31–90 days (>10 WBC/mm^3^; including traumatic LP with correction) was 15.9% (95% CI, 10.4–23.3) (7).

The etiology of sterile CSF pleocytosis in febrile infants with UTIs has not been well established. Sterile CSF pleocytosis with UTI might be caused by undiagnosed coexisting viral infections, such as enterovirus (6, 7). A few studies reported concomitant enteroviral meningitis in febrile infants with UTIs (7, 14). A multicenter retrospective study of infants aged 29–60 days with febrile UTIs showed that the proportion of the enteroviral season was higher in infants with sterile CSF pleocytosis than in those without (50.9% vs*.* 42.1%; *P* = 0.02) (13). However, consistent with our study results, several studies noted that UTI with sterile CSF pleocytosis did not occur more frequently in any particular month or during the enteroviral season (7, 14). Thus, enteroviral coinfection is insufficient as an explanation for sterile CSF pleocytosis in febrile young infants.

A plausible hypothesis for sterile CSF pleocytosis is that systemic inflammation and cytokine release resulting from bacterial UTI might cause meningeal inflammation and lead to CSF pleocytosis (7, 13, 20). Peripheral blood WBC counts, ANCs, CRP, and procalcitonin values are useful inflammatory markers that can be easily used in many clinical settings. Schnadower et al. identified the risk factors for sterile CSF pleocytosis in a large sample of febrile infants aged 29–60 days with UTI using multivariable statistical methods. Only peripheral blood WBC count was an independent risk factor (cutoff, ≥15,000/mm3; aOR, 1.97; 95% CI, 1.32–2.94), but CRP was not included in their analysis (13). Our study included WBC counts, ANCs, and CRP values as the variables in the multiple logistic regression analysis. The only independent factor associated with sterile CSF pleocytosis was CRP value (cutoff, >3.425 mg/dl; aOR, 2.77; 95% CI, 1.19–6.88). Abnormal inflammatory marker results in infants with CSF pleocytosis may cause concerns about the possibility of missing a diagnosis of bacterial meningitis even if the CSF culture is negative. However, several studies including our study showed that bacterial meningitis was rare and clinical courses and outcomes were similar regardless of sterile CSF pleocytosis in young infants >28 days old with a presumptive UTI (6, 7, 13, 19, 20, 26). These findings may support the preference for antibiotic therapy without LP in febrile young infants with positive urinalysis results beyond the neonatal period if patients have no high-risk medical history and stable vital signs.

Our study had several limitations. First, it was a retrospective single-center study, which introduces selection bias and concerns about incomplete data. Second, data on viral tests than enterovirus on CSF specimens were insufficient. Third, although based on univariable analysis, the potential predictors associated with CSF pleocytosis were focused on inflammatory markers. However, we applied criteria and definitions based on clinically accepted consensuses. While some previously published studies included patients with traumatic LP or those with negative urine culture results, our study included only patients with culture-proven UTIs and non-traumatic LPs. Furthermore, we evaluated the independent predictors associated with sterile CSF pleocytosis.

In conclusion, the overall risk of UTI and concomitant bacterial meningitis was very low in febrile infants aged 29–90 days. Sterile CSF pleocytosis was frequently observed in those with UTIs. Although coexisting sterile CSF pleocytosis with UTI may suggest a systemic inflammatory response, it did not seem to worsen clinical outcomes in healthy infants. The performance of LP in young infants with evidence of UTIs should be decided prudently, and inappropriate antibiotic therapy for sterile CSF pleocytosis should be avoided.

## Data Availability

The raw data supporting the conclusions of this article will be made available by the authors, without undue reservation.

## References

[1] Brower L Shah SS, editors. “Fever without a focus in the neonate and young infant”. In: Nelson textbook of pediatrics. Philadelphia, PA: Elsevier (2020). p. 1389–92.

[2] Powell EC Mahajan PV Roosevelt G Hoyle JD Jr Gattu R Cruz AT Epidemiology of bacteremia in febrile infants aged 60 days and younger. Ann Emerg Med (2018) 71(2):211–6. 10.1016/j.annemergmed.2017.07.488 28988964 PMC5815881

[3] Bonilla L Gomez B Pintos C Benito J Mintegi S. Prevalence of bacterial infection in febrile infant 61–90 days old compared with younger infants. Pediatr Infect Dis J. (2019) 38(12):1163–7. 10.1097/inf.0000000000002461 31568251

[4] Pantell RH Roberts KB Adams WG Dreyer BP Kuppermann N O’Leary ST Evaluation and management of well-appearing febrile infants 8–60 days old. Pediatrics. (2021) 148(2):e2021052228. 10.1542/peds.2021-052228 34281996

[5] Finkelstein Y Mosseri R Garty BZ. Concomitant aseptic meningitis and bacterial urinary tract infection in young febrile infants. Pediatr Infect Dis J. (2001) 20(6):630–2. 10.1097/00006454-200106000-00019 11419510

[6] Syrogiannopoulos GA Grivea IN Anastassiou ED Triga MG Dimitracopoulos GO Beratis NG. Sterile cerebrospinal fluid pleocytosis in young infants with urinary tract infection. Pediatr Infect Dis J. (2001) 20(10):927–30. 10.1097/00006454-200110000-00003 11642625

[7] Adler-Shohet FC Cheung MM Hill M Lieberman JM. Aseptic meningitis in infants younger than six months of age hospitalized with urinary tract infections. Pediatr Infect Dis J. (2003) 22(12):1039–42. 10.1097/01.inf.0000100576.99266.07 14688561

[8] Goldman RD Matlow A Linett L Scolnik D. What is the risk of bacterial meningitis in infants who present to the emergency department with fever and pyuria? Cjem. (2003) 5(6):394–9. 10.1017/s1481803500008630 17466129

[9] Vuillermin PJ Starr M. Investigation of the rate of meningitis in association with urinary tract infection in infants 90 days of age or younger. Emerg Med Australas. (2007) 19(5):464–9. 10.1111/j.1742-6723.2007.01001.x 17919220

[10] Shah SS Zorc JJ Levine DA Platt SL Kuppermann N. Sterile cerebrospinal fluid pleocytosis in young infants with urinary tract infections. J Pediatr. (2008) 153(2):290–2. 10.1016/j.jpeds.2008.02.044 18639733

[11] Yam AO Andresen D Kesson AM Isaacs D. Incidence of sterile cerebrospinal fluid pleocytosis in infants with urinary tract infection. J Paediatr Child Health. (2009) 45(6):364–7. 10.1111/j.1440-1754.2009.01502.x 19490407

[12] Paquette K Cheng MP McGillivray D Lam C Quach C. Is a lumbar puncture necessary when evaluating febrile infants (30–90 days of age) with an abnormal urinalysis? Pediatr Emerg Care. (2011) 27(11):1057–61. 10.1097/PEC.0b013e318235ea18 22068068

[13] Schnadower D Kuppermann N Macias CG Freedman SB Baskin MN Ishimine P Sterile cerebrospinal fluid pleocytosis in young febrile infants with urinary tract infections. Arch Pediatr Adolesc Med. (2011) 165(7):635–41. 10.1001/archpediatrics.2011.104 21727275

[14] Doby EH Stockmann C Korgenski EK Blaschke AJ Byington CL. Cerebrospinal fluid pleocytosis in febrile infants 1−90 days with urinary tract infection. Pediatr Infect Dis J. (2013) 32(9):1024–6. 10.1097/INF.0b013e31829063cd 23584580 PMC3755104

[15] Thomson J Cruz AT Nigrovic LE Freedman SB Garro AC Ishimine PT Concomitant bacterial meningitis in infants with urinary tract infection. Pediatr Infect Dis J. (2017) 36(9):908–10. 10.1097/inf.0000000000001626 28472006

[16] Young BR Nguyen THP Alabaster A Greenhow TL. The prevalence of bacterial meningitis in febrile infants 29−60 days with positive urinalysis. Hosp Pediatr. (2018) 8(8):450–7. 10.1542/hpeds.2017-0254 29987127

[17] Nugent J Childers M Singh-Miller N Howard R Allard R Eberly M. Risk of meningitis in infants aged 29–90 days with urinary tract infection: a systematic review and meta-analysis. J Pediatr. (2019) 212:102–10.e5. 10.1016/j.jpeds.2019.04.053 31230888

[18] Burstein B Sabhaney V Bone JN Doan Q Mansouri FF Meckler GD. Prevalence of bacterial meningitis among febrile infants aged 29−60 days with positive urinalysis results: a systematic review and meta-analysis. JAMA Netw Open. (2021) 4(5):e214544. 10.1001/jamanetworkopen.2021.4544 33978724 PMC8116985

[19] Mahajan P VanBuren JM Tzimenatos L Cruz AT Vitale M Powell EC Serious bacterial infections in young febrile infants with positive urinalysis results. Pediatrics. (2022) 150(4):e2021055633. 10.1542/peds.2021-055633 36097858 PMC9648158

[20] Rafferty A Drew RJ Cunney R Bennett D Marriott JF. Infant escherichia coli urinary tract infection: is it associated with meningitis? Arch Dis Child. (2022) 107(3):277–81. 10.1136/archdischild-2021-322090 34285001

[21] Scarfone R Murray A Gala P Balamuth F. Lumbar puncture for all febrile infants 29−56 days old: a retrospective cohort reassessment study. J Pediatr. (2017) 187:200–5.e1. 10.1016/j.jpeds.2017.04.003 28526220 PMC5540147

[22] Kasmire KE Hoppa EC Patel PP Boch KN Sacco T Waynik IY. Reducing invasive care for low-risk febrile infants through implementation of a clinical pathway. Pediatrics. (2019) 143(3):e20181610. 10.1542/peds.2018-1610 30728272

[23] Jerardi KE Jackson EC. “Urinary tract infection”. In: Nelson Textbook of pediatrics. Philadelphia, PA: Elsevier (2020). p. 2789–98.

[24] Byington CL Kendrick J Sheng X. Normative cerebrospinal fluid profiles in febrile infants. J Pediatr. (2011) 158(1):130–4. 10.1016/j.jpeds.2010.07.022 20801462 PMC2994954

[25] Dieckmann RA Brownstein D Gausche-Hill M. The pediatric assessment triangle: a novel approach for the rapid evaluation of children. Pediatr Emerg Care. (2010) 26(4):312–5. 10.1097/PEC.0b013e3181d6db37 20386420

[26] Kuppermann N Dayan PS Levine DA Vitale M Tzimenatos L Tunik MG A clinical prediction rule to identify febrile infants 60 days and younger at low risk for serious bacterial infections. JAMA Pediatr. (2019) 173(4):342–51. 10.1001/jamapediatrics.2018.5501 30776077 PMC6450281

