# Supplementary material for: Clinical characteristics and outcomes in febrile infants aged 29–90 days with urinary tract infections and cerebrospinal fluid pleocytosis
Source: Front Pediatr. 2023 May 30;11:1196992. doi: 10.3389/fped.2023.1196992 (PMC10267820; doi:10.3389/fped.2023.1196992)
Supplement: Supplementary file 1 [file Datasheet1.pdf]

# *Supplementary Material*

# Clinical characteristics and outcomes in febrile infants aged 29 to 90 days with urinary tract infections and cerebrospinal fluid pleocytosis

**Ga Won Moon, Donghyun Shin, Young Mi Kim, Soo-Han Choi***

*** Correspondence:** Soo-Han Choi: soohan_choi@pusan.ac.kr

## 1 Supplementary Figures and Tables

### 1.1 Supplementary Figures

**(A) White blood cell count** **(B) Absolute neutrophil count**

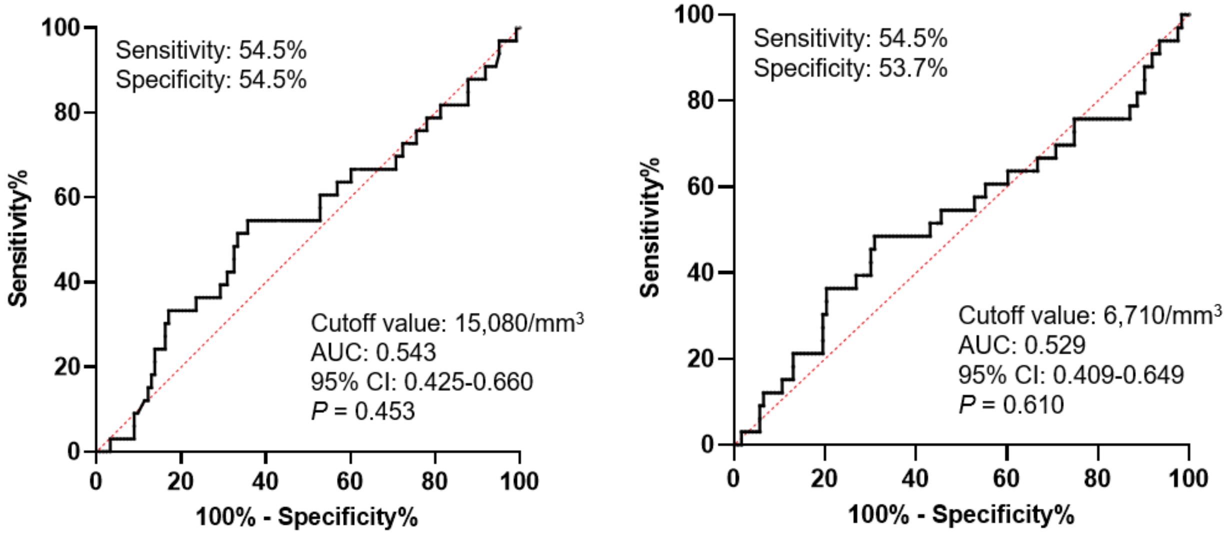

**(C) Platelet count**

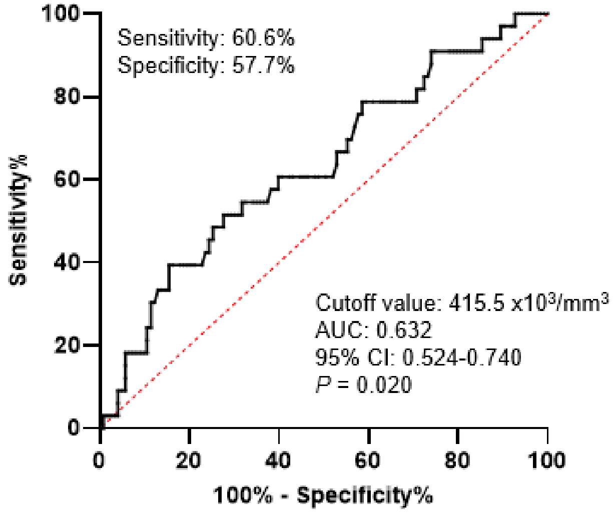


**(D) C-reactive protein**

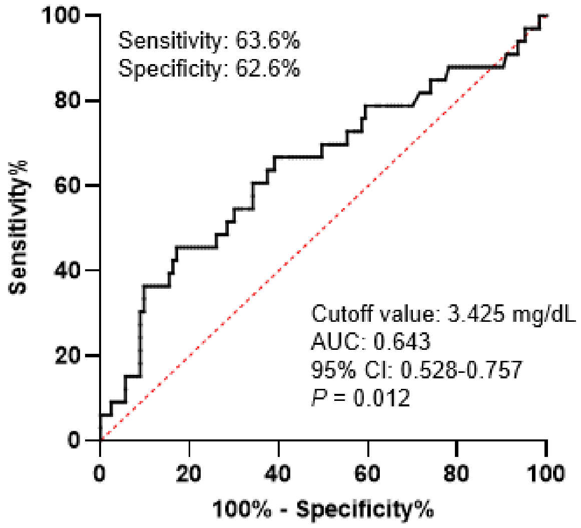


**Supplementary Figure 1.** The Laboratory cutoff values that maximized the area under the receiver operating characteristic curve (AUC).

## 1.2 Supplementary Tables

**Supplementary Table 1. Association of CSF white blood cell counts with the clinical parameters**

| **Spearman's correlation analysis** | | | |
|---|---|---|---|
| Variables | Spearman r | 95% CI | *P*-value |
| Age | -0.008 | -0.239 to 0.082 | 0.319 |
| Time from fever onset to the hospital visit | 0.155 | -0.007 to 0.309 | 0.054 |
| White blood cell | 0.133 | -0.030 to 0.288 | 0.099 |
| Absolute neutrophil count | 0.078 | -0.084 to 0.237 | 0.331 |
| Platelet | 0.153 | -0.009 to 0.307 | 0.057 |
| C-reactive protein | 0.234 | 0.075 to 0.381 | 0.003 |

CI, confidential interval

**Supplementary Table 2. Characteristics of febrile infants aged 29-60 days with UTI**

| | Total (N = 72) | Well-appearing term infants (n = 30) | Others [a] (n = 42) | *P*-value |
|---|---|---|---|---|
| Fever of > 38.5 ℃ (%) | 37 (51.4) | 12 (40.0) | 25 (59.5) | 0.151 |
| CRP ≥20 mg/L (%) | 45 (62.5) | 19 (63.3) | 26 (61.9) | >0.999 |
| ANC > 5200 per $mm^3$ (%) | 47 (65.3) | 20 (66.7) | 27 (64.3) | >0.999 |
| Abnormal inflammatory markers in the AAP guidelines [b] (%) | 31 (43.1) | 12 (40.0) | 19 (45.2) | 0.810 |
| CSF pleocytosis (%) | 3 (18.1) | 5 (16.7) | 8 (19.0) | >0.999 |
| Bacteremia (%) | 3 (4.2) | 1 (3.3) | 2 (4.8) | >0.999 |

[a] Not well-appearing or preterm infants

[b] Fever of > 38.5 ℃ in combination with CRP ≥20 mg/L or ANC > 5200 per $mm^3$

UTI, urinary tract infection; CRP, C-reactive protein; ANC, absolute neutrophil count; AAP, American Academy of Pediatrics; CSF, cerebrospinal fluid
